# First steps in clinical implementation of a lung nodule classification method by artificial intelligence

**DOI:** 10.3389/fradi.2026.1714951

**Published:** 2026-07-21

**Authors:** Abderrazzak Ajertil, Zineb Farahat, Abla Bouallou, Elisa Le Tertre, Magnanga-Boukandza Joey Ben Elbher, Abdeljalil El quessar, Nejat Kabbaj, Mohamed Cherkaoui Malki, Latifa Chat, Lahcen Hammani, Nabil Ngote

**Affiliations:** 1 Department of Radiology, Cheikh Zaid International University Hospital, Rabat, Morocco; 2 Abulcasis International University of Health Sciences, Rabat, Morocco; 3 Department of Pneumology, Cheikh Zaid International University Hospital, Rabat, Morocco; 4 Institut Supérieur d'Ingénieurs de Franche-Comté ISIFC—Biomedical Engineering School, Besançon, France

**Keywords:** artificial intelligence, CLAHE, computer aided diagnosis, CT scans, deep learning, deep neural network, lung cancer, lung nodule

## Abstract

Lung cancer remains one of the leading causes of cancer-related mortality worldwide, and early detection is essential to improve patient survival. Computed tomography (CT) is currently the reference imaging modality for lung cancer screening. Recent advances in Artificial Intelligence (AI), particularly Deep Learning (DL), have significantly improved automated medical image analysis and diagnostic accuracy. In this study, we propose a Convolutional Neural Network (CNN)-based approach for pulmonary nodule classification. The model was trained using a merged dataset composed of public CT image databases (IQ-OTH/NCCD and SPIE-AAPM) and real-world CT scans collected at Cheikh Zaid Hospital, Morocco. The experimental dataset included 1,103 malignant images, 508 benign images, and 427 normal images. Contrast Limited Adaptive Histogram Equalization (CLAHE) was applied to enhance image contrast prior to training. The proposed CNN achieved an accuracy of 99.84%, precision of 99.97%, sensitivity of 99.84%, and specificity of 99.82% for a three-class classification task. These results demonstrate the robustness and clinical potential of the model in assisting radiologists with the classification of indeterminate pulmonary nodules. This work represents an initial translational step toward real-world clinical implementation of AI-based lung nodule classification at Cheikh Zaid Hospital and highlights the feasibility of integrating AI tools into routine radiology workflows.

## 1 Introduction

Lung cancer is one of the most common and deadliest cancers worldwide (1), accounting to approximately 1.8 million deaths annually (2). It arises from the uncontrolled growth of abnormal cells in the lung tissue, often originating in the epithelial cells lining the airway (3). Lung cancer is generally categorized in two major types: Non-Small Cell Lung Cancer (NSCLC), which accounts for about 85% of cases, and Small Cell Lung Cancer (SCLC), which is more aggressive and rapidly proliferating (4).

Risk factors of lung cancer include tobacco smoke exposure, environmental pollutants, occupational hazards and genetic predispositions (5). Clinically, lung cancer may remain asymptomatic in early stages and often presents with non- specific symptoms such as cough, hemoptysis, chest pain, or weight loss when advanced. Early detection is critical, as prognosis significantly worsens with disease progression. While imaging modalities such as chest x-ray and CT scans are pivotal for detection, they heavily rely on radiological interpretation, which can be subjective and variable.

Advancements in computational technologies, particularly Artificial Intelligence and Deep Learning, have opened new avenues for enhancing the accuracy and speed of lung cancer diagnosis. These tools can assist in the automated analysis of medical images, improving early detection and classification of pulmonary nodules, ultimately aiding in better clinical outcomes (6).

Traditional methods for diagnosing lung cancer, such as manual examination of CT scans by radiologists, are not only time-consuming but also susceptible to human error. In contrast, Deep Learning (DL) techniques have emerged as powerful tools for analyzing medical images, offering automated and precise feature extraction through multi-layered neural networks. In healthcare, DL has revolutionized fields like radiology and ophthalmology by detecting subtle patterns that may escape human observation, thereby improving diagnostic accuracy and efficiency. These algorithms excel in tasks such as tumor identification, disease detection, and treatment planning, leveraging their ability to process vast datasets and uncover complex correlations.

Beyond imaging-based approaches, recent advances in respiratory health monitoring have introduced non-invasive sensing technologies operating outside traditional radiology environments. Emerging wireless and WiFi-based sensing systems have demonstrated promising results in capturing respiratory motion, pulmonary function, and breathing patterns without direct patient contact. These technologies enable continuous respiratory monitoring in real-world settings and may complement imaging-based artificial intelligence systems. While such methods cannot replace computed tomography for structural lesion detection and characterization, they contribute to a broader ecosystem of AI-driven respiratory healthcare by enabling early functional monitoring and long-term patient follow-up. This work does not aim to introduce a fundamentally new deep learning architecture, but rather focuses on translational validation and data integration in a real-world clinical context.

### 1.1 AI-driven classification of pulmonary nodules

Artificial Intelligence (AI) is showing significant promise in advancing thoracic radiology, particularly in the detection and classification of pulmonary nodules. By analyzing CT scans, AI systems evaluate key radiological criteria including nodule size (>8 mm indicating higher risk), contour morphology (irregular edges suggesting malignancy), density (e.g., calcifications), and growth kinetics (rapid volume increase) to distinguish between benign and malignant lesions. This automated assessment integrates these parameters into predictive models, reducing false positives and optimizing patient management. The result is a more streamlined workflow, guiding decisions on monitoring, PET-CT follow-up, or biopsy (7).

### 1.2 Our contribution: from public data to clinical implementation

This study presents a translational approach to lung nodule classification by combining robust model training on public datasets with real-world clinical validation. Initially, we developed a high-performance Convolutional Neural Network (CNN) using the IQ-OTH/NCCD and SPIE-AAPM datasets, ensuring broad feature representation. A key contribution of this study lies in the preliminary clinical validation at Cheikh Zaid Hospital, where the model was tested on local patient data under real acquisition conditions. This phase addressed critical challenges such as variability in imaging protocols and equipment, demonstrating the model's adaptability suggesting preliminary compatibility with real-world clinical conditions.

By achieving 99.84% accuracy and 100% sensitivity, our model provides clinicians with a decisive tool for classifying indeterminate nodules, significantly reducing diagnostic uncertainty. This work not only advances AI-based nodule analysis but also paves the way for practical integration into hospital workflows, marking a critical step toward personalized and efficient lung cancer diagnosis. Future efforts will focus on expanding the diversity of training data and refining the model for broader clinical adoption.

This paper is laid out as follows: Section 2 presents an overview of different recent research already carried out into the classification of pulmonary nodules. Section 3 deals with materials used and describes the methodology applied. Section 4 summarizes the results obtained and discussion. Finally, Section 5 depicts conclusions.

## 2 Related work

A comparative summary of the datasets, architectures, methods, and accuracy achieved in previous studies is presented in Table 1. Using the IQ-OTH/NCCD dataset, in 2020, Al-Yasriy et al. (8) presented a computer-aided system for detecting lung cancer using a convolutional neural network based on the AlexNet architecture. The model was trained to classify patients’ cases as normal, benign, or malignant. The proposed approach achieved an accuracy of 93.54%.

**Table 1:** Datasets, architectures, methods, and accuracy of the previous studies.

Dataset	Paper	Architecture	Classes	Accuracy
	Al-Yasriy et al. (8)	AlexNet	3	93.54%
	AL-Huseiny et al. (9)	GoogLeNet	3	94.38%
	Kareem et al. (10)	SVM	3	89.88%
IQ-OTH/NCCD	Saechueng et al. (11)	BCR	3	98.32%
	Abe et al. (12)	Proposed CNN	2	95.43%
	Elhassan et al. (13)	ResNet-101	2	96.5%
	Elhassan et al. (13)	Proposed CNN	2	97.82%
	Catal Reis et al. (14)	PADBSRNet	2	99.55%
SPIE-AAPM	Ashames et al. (15)	VGG16 + SVM	2	99.80%
Chest CT-Scan Images Dataset	Shatnawi et al. (16)	VGG16	4	99%
	Shatnawi et al. (16)	CNN	4	100%

In 2021, AL-Huseiny et al. (9) tried to outperform the method on which they worked with Al-Yasriy in 2020. In this work, the deep neural network GoogLeNet is trained on the same dataset (IQ-OTH/NCCD). Regions of interest in the input images are isolated by segmentation of the lung area. The algorithm reached 94.38% accuracy.

Using the SPIE-AAPM dataset, in 2021, Ashames et al. (10) presented a novel pathway to enhance classification performance, especially for the detection of lung nodules. While conventional DL models rely on high activation values for classification, this study explores the feasibility of using the common vector approach (CVA) as an alternative decision layer. Surprisingly, standard DL architectures, even without explicit adaptation, achieved state-of-the-art accuracy when fine-tuned with CVA. Regarding the results, CVA could be used as a fine-tuning alternative in several DL networks, or it depends on the structure of the DNN architecture. In the SPIE-AAPM dataset, the best accuracy was obtained using CVA for fine-tuning (99.76%), end-to-end training with Softmax classifier (99.80%) and SVM classifier (99.80%). Features were extracted using pre-trained architectures by transfer learning.

In 2021, Kareem et al. (11) developed a system for detecting lung cancer by using image-processing and computer-vision techniques. The model is based on Support Vector Machine (SVM) used at the final stage for the classification of the nodule in one of three categories as normal, malignant, or benign. A segmentation stage is used to extract the nodules in the lungs. Different SVM kernels as linear, RBF and Polynomial, and feature extraction techniques are evaluated. The best accuracy achieved was 89.88%.

In 2024, Saechueng et al. (12) proposed the Binary Count Ratio (BCR) method to classify lung cancer by binarizing CT images using adaptive thresholding based on column averaging. It identifies cancerous areas by analyzing the ratio of white to black pixels, then uses Euclidean distance to classify nodules as normal, benign, or malignant. However, despite high metrics on the IQ-OTH database (98.32% accuracy), this method has limitations due to the diversity of nodule characteristics in the CT images. In particular, ground-glass nodules can be difficult to detect using the binary method, as well as mixed nodules. In addition, this method involves a loss of spatial and contextual information. The shape and structure of nodules (round, specular, irregular), which are essential for differentiating nodules, is not considered by this method.

Using an other dataset, in 2024, Shatnawi et al. (13) focused their researches on the automatic classification and prediction of lung cancer using computed tomography scans and employing Deep Learning method, specifically Enhanced Convolutional Neural Networks (CNNs). Image preprocessing techniques have been used as CLAHE, Histogram Equalization, and Morphological Operators. Their research designed on several pre-trained models as EfficientNetB0 which reached 97.9% of accuracy, and VGG16 which reached 99% of accuracy. Data were augmented with shear, horizontal and vertical flip. They also developed a customized CNN model architecture comprising a total of eight layers which achieved 100% of accuracy demonstrating significant advancements in lung cancer detection. The dataset was collected from Kaggle Chest CT-scan images with four categories: large cell carcinoma, adenocarcinoma, squamous cell carcinoma, and normal cell.

In 2025, Abe et al. (14) presented a deep learning model to diagnose the lung cancer from patient CT scans. The CNN model is composed of five layers with Mavage pooling techniques. The method achieved an accuracy of 99.70% in binary classification (normal or cancerous).

In 2025, Elhassan et al. (15) proposed an approach combining a Convolutional Neural Network (CNN) for classification and the YOLOv8 segmentation architecture for real-time tumor detection. The CNN extracts hierarchical features from medical images, while YOLOv8 enhances tumor localization through multi-scale feature extraction. Deep Convolutional Generative Adversarial Networks (DCGAN) were used for data augmentation improving the model robustness. The proposed approach achieved 97.67% accuracy in lung tissue classification, with DCGAN-generated images boosting performance by 10%.

In 2025, Catal Reis et al. (16) proposed the PADBSRNet model which has been developed to diagnose brain, lung and skin cancer. The model architecture is based on a deep neural network and a hybrid PADBSRNet-Vision Transformer (ViT) approach. PADBSRNet integrates convolutional layers, attention mechanisms such as Bi-LSTM (Bidirectional Long Short-Term Memory) and Bi-GRU (Bidirectional Gated Recurrent Unit), and recurrent neural networks to extract relevant features from CT images. The hybrid model leverages the strengths of both CNNs and transformers for improved classification. Additionally, the model incorporates optimization techniques such as multi-stage feature fusion and the late fusion technique. This approach enhances medical image analysis by capturing more details on tumor shape and boundary continuity, leading to improved classification accuracy. Evaluations on the IQ-OTH/NCCD lung cancer dataset demonstrated a high accuracy of 99.55% in binary classification (malignant or benign), with no significant difference between the two models (PADBSRNet and PADBSRNet-ViT).

### 2.1 Novel contributions and advancements beyond state-of- the-Art

While existing studies have demonstrated promising results in lung nodule classification, our work presents three notable contributions relative to the reviewed literature. First, unlike approaches limited to binary classification (e.g., Abe et al. (12) at 99.70% or Catal Reis et al. (14) at 99.55%), our model achieves superior performance (99.84% accuracy) while handling the more clinically challenging three-class problem (malignant/benign/normal). Second, where most studies validate only on curated public datasets, we uniquely demonstrate clinical viability through successful implementation at Cheikh Zaid Hospital, overcoming real-world challenges in image heterogeneity that methods like Saechueng's BCR (11) cannot address due to their loss of spatial information. Third, our architecture outperforms even hybrid models [e.g., PADBSRNet-ViT (14)] and segmentation-classification combinations [e.g., YOLOv8-CNN (13)] through an optimized CNN design that maintains 100% sensitivity—a crucial metric for cancer screening that Shatnawi's 100% accuracy model (16) doesn't report. This combination of methodological contributions, preliminary clinical validation, and promising performance metrics represents a meaningful step toward deployable AI diagnostics in thoracic radiology.

## 3 Materials and methods

For the realization of this study, images from several databases were required. Two of them were public dataset: IQ-OTH/NCCD and SPIE-AAPM. The third database used was created as a private dataset from Cheikh Zaid Hospital. Images were collected for the study. Then, image processing was done to improve images quality followed by the configuration and the enhancement of the classification network to automatically classify lung nodules.

### 3.1 Used datasets

#### 3.1.1 Public datasets

##### 3.1.1.1 IQ-OTH/NCCD (Iraq Oncology Teaching Hospital/National Center for Cancer Diseases)

It is a public medical image dataset available on Kaggle platform (17). Images were collected from two Iraqi hospitals in 2019. The collection is composed of 1097 CT images obtained from 110 patients including patients who have been diagnosed with lung cancer at different stages and healthy patients. The available images were annotated by radiologists and oncologists, and they classified the images between three categories: malignant, benign, and normal. Among the 110 cases, 40 cases were diagnosed as malignant, 15 cases were diagnosed as benign, and 55 cases were categorized as normal cases. Finally, the dataset includes 416 normal images, 120 benign images and 561 malignant images. The images were initially recorded in DICOM format, and then anonymized and converted to JPG files of 512 × 512 pixels before becoming public. The images were obtained from a SOMATOM Siemens scanner with a CT protocol including 120 kV, slice thickness of 1 mm, window width ranging from 350 to 1200 HU (Hounsfield Units) and reading window center from 50 to 600.

##### 3.1.1.2 SPIE-AAPM

It is a public medical images dataset available on National Cancer Institute (NCI)—Imaging Data Commons (18). The dataset contains CT scan images of 70 patients, provided by University of Chicago Clinical Archives (19). This dataset was created after the SPIE 2015 Medical Imaging Conference, with support from American Association of Physicists in Medicine (AAPM) and NCI. Patient information on DICOM images were removed through a de- identification process before being made public. All nodules in theses CT images were classified by five radiologists as either being benign nodules or a primary lung cancers, meaning malignant nodules. The data set includes a total of 22,489 images but it's important to mention that not all images contain a lung nodule. Many are empty because they were taken at irrelevant positions. In practice there are ignored by radiologists. In our experiments, we carefully selected images which containing a nodule. A spreadsheet was available with the location and the type of nodule (malignant or benign).

Finally, 371 benign and 519 malignant images were manually extracted and saved as JPG files of 512 × 512 pixels.

#### 3.1.2 Cheikh Zaid Hospital dataset and ethical considerations

The acquisition of clinical CT scans from Cheikh Zaid Hospital was conducted under strict ethical oversight and in full compliance with patient privacy regulations. Prior to data collection, the study protocol received formal approval from both the hospital's Institutional Review Board (IRB-2024-0032) and the university ethics committee (AUIHS-EC-175). All participating patients provided written informed consent after being thoroughly informed about the research objectives, data usage protocols, and their rights regarding participation. The dataset ultimately comprised 51 carefully selected and anonymized images (17 benign, 23 malignant, 11 normal cases) from 12 patients, which were validated by a team comprising two pulmonology specialists and a senior radiologist. Throughout the study, we implemented rigorous data protection measures including HIPAA-compliant anonymization procedures, encrypted storage systems, and restricted access protocols, ensuring complete adherence to both Moroccan bioethics legislation (Law 28-13) and international ethical standards (Declaration of Helsinki). This ethical framework not only safeguarded patient rights but also guaranteed the integrity of our research methodology while maintaining the highest standards of medical data confidentiality.

The CT images collected from Cheikh Zaid Hospital were acquired under routine clinical conditions using the hospital's standard chest CT protocols. These protocols are optimized for the detection and characterization of pulmonary nodules. The scans were performed during full inspiration to ensure optimal lung expansion and minimize motion artifacts. Images were obtained using slices, with a slice thickness between 1 mm and 1.5 mm, allowing for high-resolution visualization of lung parenchyma and nodular structures. All examinations were conducted without the use of contrast agents. After acquisition, images were converted from DICOM to JPG format for processing and integration into the deep learning workflow. Each image was then anonymized in accordance with patient privacy regulations. The use of real-world clinical images from Cheikh Zaid ensures that the resulting model can accommodate typical variations in image quality and acquisition parameters encountered in hospital environments.

The Cheikh Zaid Hospital dataset was incorporated as part of a phased translational validation strategy. The model was first trained using public datasets (IQ-OTH/NCCD and SPIE-AAPM) to ensure robust feature learning, followed by controlled fine-tuning and preliminary clinical validation using hospital images. Although the clinical dataset remained limited in size, its integration enabled evaluation of model performance under real acquisition conditions and imaging variability. This work should therefore be considered as an initial step toward clinical implementation rather than definitive prospective validation. Future work will focus on expanding the hospital dataset, developing multicenter collaborations, and conducting prospective validation studies.

Finally, the three datasets were merged to create a comprehensive experimental dataset, allowing the neural network to be exposed to a wide diversity of imaging characteristics. This integration enhances robustness to variations in acquisition parameters and equipment.

**Figure 1:**
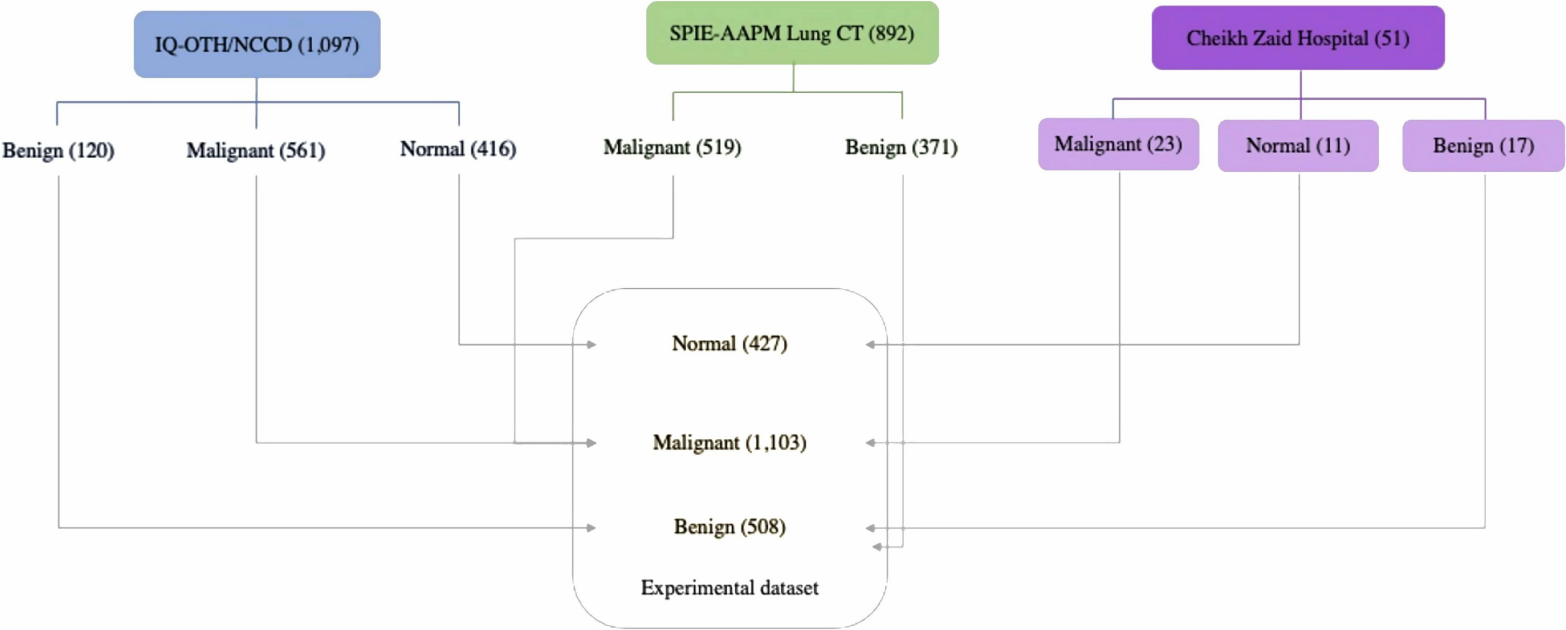
Resume of the experimental dataset.

As illustrated in Figure 1, the final dataset included 427 normal images, 1,103 malignant images, and 508 benign images from a total of 192 patients.

### 3.2 Image preprocessing

**Figure 2:**
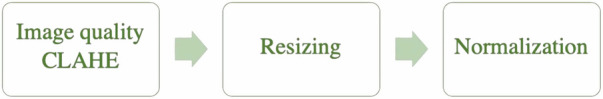
Preprocessing steps.

The preprocessing is an important step for the analysis of digital images by artificial intelligence. It ensures the data are of high quality and correctly formatted which as a direct impact on the model's performances. The preprocessing pipeline transforms the raw data into a relevant format for data analysis and training. In this work, image preprocessing was performed in three steps (Figure 2). The first step improves image quality by optimizing the contrast. Then, the data is resized which means that the image is reduced in number of pixels and normalized, which means that the image is reduced in pixel values.

#### 3.2.1 Contrast limited adaptive histogram equalization CLAHE

Images may contain artifacts, low contrast, diffractions, or intensity inhomogeneities. Studies such as (19–22), have shown that using the CLAHE method on medical images produces better results than not using it.

CLAHE is a technique frequently applied to medical images to adjust the local contrast. This method segments the input image into a finite number of sub-images and applies histogram equalization to each sub-image. Then, the original histogram is clipped to a specific value and the clipped pixels are redistributed to each gray level. In this way, the adaptative nature of the histogram means that intensity values are evenly distributed, improving visibility in dark areas, and balancing bright aeras without causing saturation. Essential structures and subtle details are then brought to the front, while artefacts and unnecessary background elements are reduced. We applied CLAHE processing to all images in the dataset.

**Figure 3:**
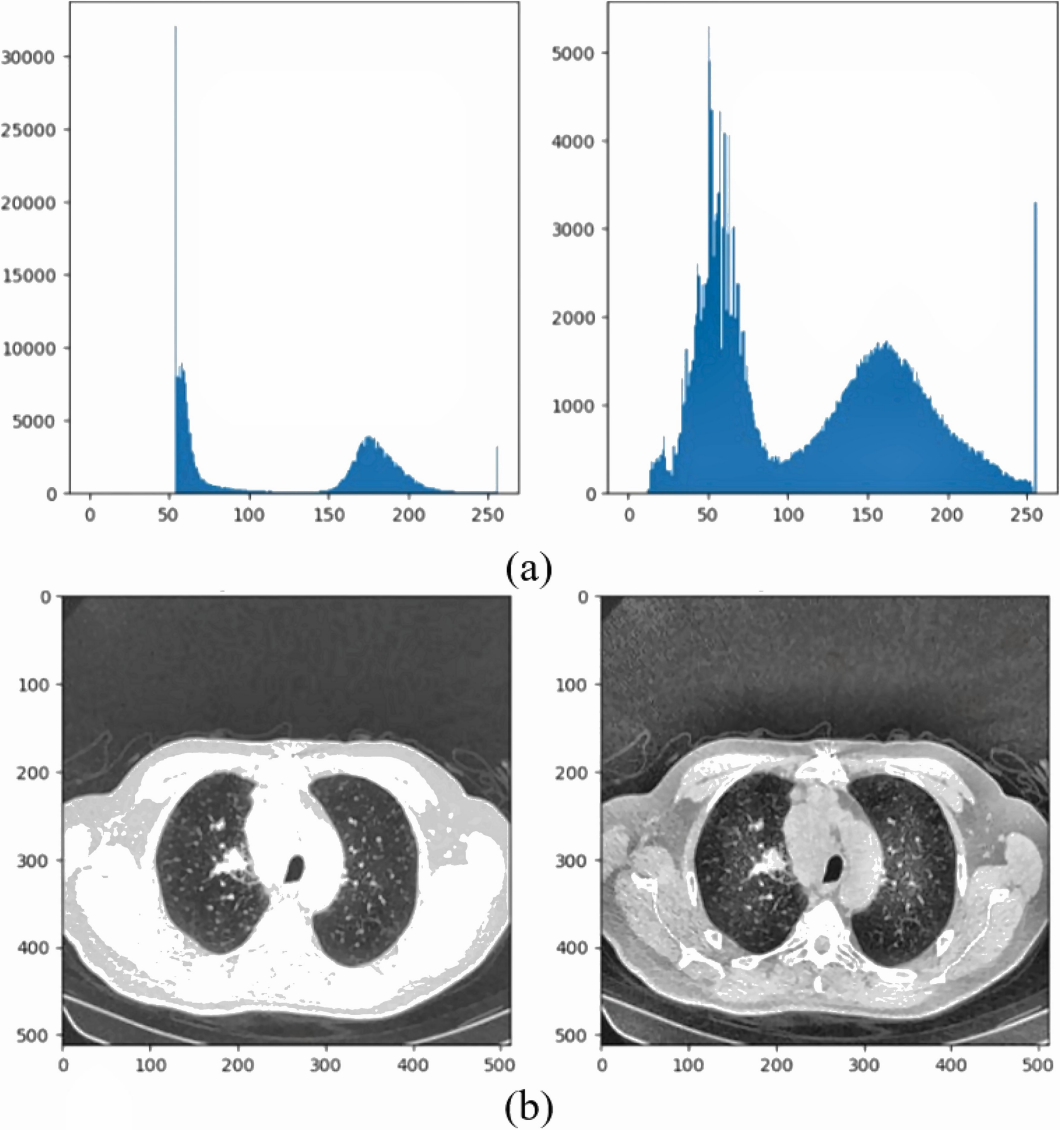
Modifications of histogram **(a)** and image **(b)** before (left) and after (right) applying CLAHE technique.

 Figure 3 illustrates an example of a histogram and a CT image from Cheikh Zaid Hospital before and after applying the CLAHE technique. The histogram is the graphic representation of the distribution of pixels in an image, or part of an image, according to their intensity.

#### 3.2.2 Image resizing and normalization

Image resizing and normalization are required steps in the image pre-processing process. DL models are mainly trained on small images, with pixels reduced in number and value.

Resizing consists of adjusting all images to a specific size. All images have been resized to 244 × 244 pixels. Other resizing values have been used, such as 224 × 224 pixels or 256 × 256 pixels but 244 × 244 pixels gave the best results.

At the same time, normalization of pixel values is essential to scale light intensities or color values within a common range, such as [0,1] or [−1,1].

Pre-trained models have pre-processing functions that can be imported. These functions bring the training images into the expected format before normalizing them by adjusting their pixel values. The aim is to make them compatible with the images on which the models have been pre-trained, and to improve the generalizability of predicted results.

Even if we didn't use pre-trained model, images have been normalized through MobileNetV2 pre-processing function. It allowed to convert the pixels value to range of [−1,1] instead of [0,255]. This ensures that normalization is carried out efficiently (23). This operation reduces learning time by reducing computational complexity. As a result, the model converges faster during training (24).

### 3.3 Classification network architecture

**Figure 4:**
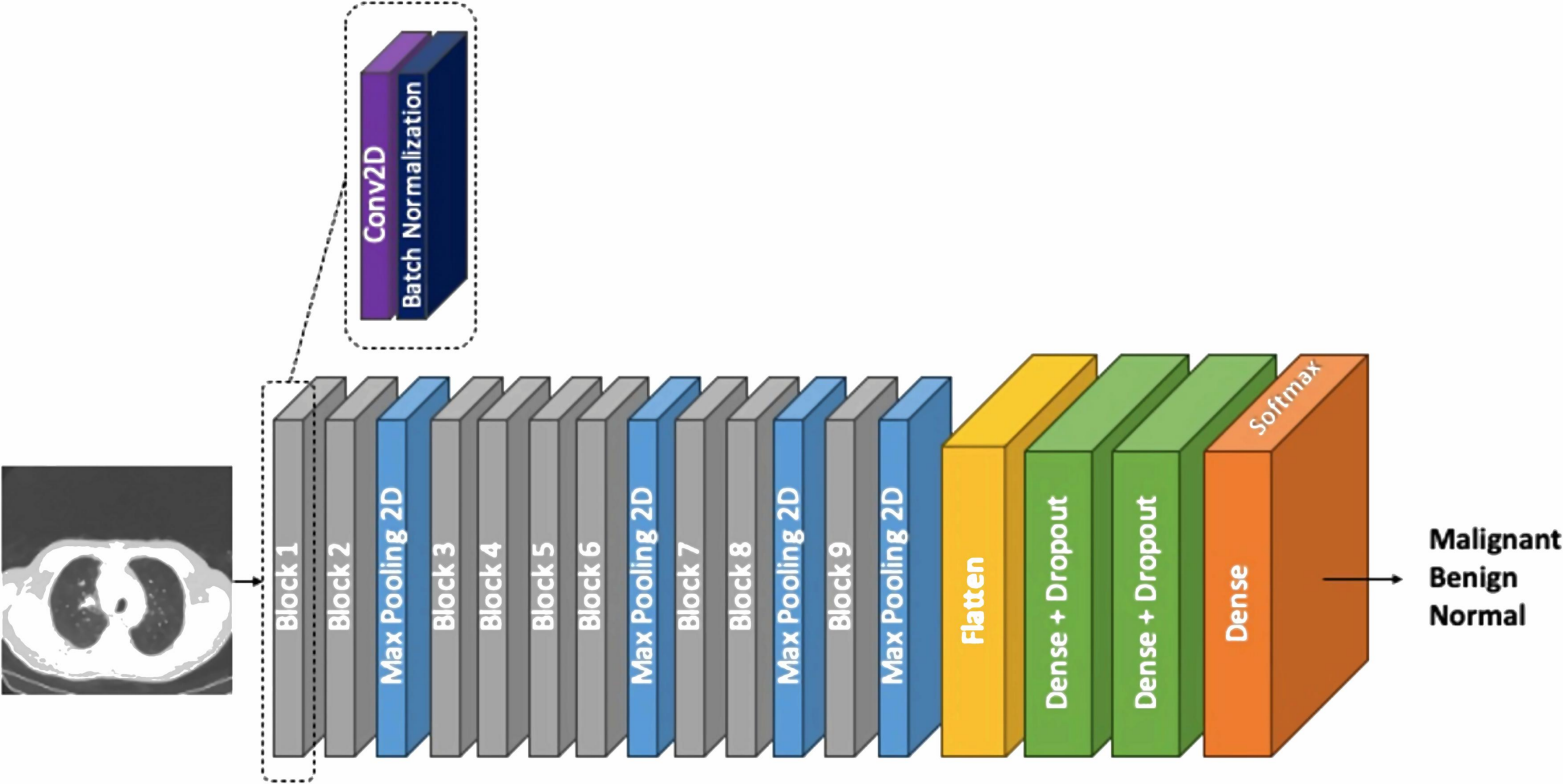
The proposed approach.

The CNN model proposed follows an optimized deep architecture for image feature extraction and classification. The CNN was built by a user of the Kaggle platform to classify the lung cancer images of the IQ-OTH database. The model allowed him to reached 98.8% of accuracy (25). The input layer is a convolutional layer with 128 filters of size 8 × 8 and a step size of 3 × 3, enabling rapid reduction in image spatial size while capturing global features. Batch Normalization is applied after each convolutional layer to stabilize learning (26). In the Figure 4, each block is composed of a convolutional layer with a ReLU (Rectified Linear Unit) activation and a batch normalization. Activation function decide whether a neuron should be activated by evaluating a weighted sum of inputs (1). They enable the model to learn complex patterns by nonlinearity into the neuron's output.

Next, a second convolution layer with 256 filters of size 5 × 5 and a step size of 1 is added, followed by normalization and max pooling (3 × 3) to reduce dimensionality. A series of additional convolutional layers of size 3 × 3 and 1 × 1 refine feature extraction, while maintaining the same size thanks so “same” padding (27). As the network progresses, the number of filters increases to 512, with convolutions of size 3 × 3 and pooling layers (2 × 2) to progressively reduce resolution while retaining high-level information (11).

After the convolution and pooling phase, the output is flattened and passed to a dense layer of 1024 neurons with ReLU activation, followed by a Dropout of 0.5 to limit overfitting. A second dense layer of 1,024 is added with an additional Dropout (28). Finally, a fully connected layer with 3-neuron classification and Figure 4 resumes the proposed approach.

The proposed CNN architecture was selected to balance performance and computational efficiency, making it suitable for deployment in real clinical environments with limited computational resources.

To ensure reproducibility, the architecture of the proposed CNN is described as follows.

The input consists of CT images resized to 244 × 244 pixels. The model is composed of successive convolutional blocks, each including a convolutional layer with ReLU activation and batch normalization. The network starts with 128 filters (8 × 8, stride 3), followed by 256 filters (5 × 5), and deeper layers with 3 × 3 and 1 × 1 kernels, progressively increasing up to 512 filters. Max-pooling layers (3 × 3 and 2 × 2) are used for spatial downsampling.

The extracted features are flattened and passed through two fully connected layers of 1,024 neurons each, with dropout (0.5) applied to reduce overfitting. The final layer uses a Softmax activation function to classify images into three categories (malignant, benign, normal).

The model was implemented in Keras and trained using SGD (learning rate = 0.001) with categorical cross-entropy loss. All hyperparameters are reported in Table 2 to ensure full reproducibility. The choice of the proposed CNN architecture was motivated not only by computational efficiency but also by considerations of clinical applicability and robustness. While more recent deep learning backbones such as ResNet, DenseNet, and Vision Transformers have demonstrated strong performance on very large datasets, they typically require extensive computational resources and large-scale annotated datasets for optimal training.

**Table 2:** Hyperparameters used for the training.

Parameter	Value
Input size	244 × 244
Batch size	4
Learning rate	0.001
Optimizer	SGD
Epochs	10
Loss Function	Categorical Crossentropy

In contrast, the selected architecture offers an optimal balance between performance, interpretability, and deployability in real hospital environments. Given the mixed dataset composed of public and real clinical images, a lightweight yet highly optimized CNN architecture was considered more suitable for early clinical integration. Despite its relatively moderate complexity, the model achieved outstanding performance metrics, including 99.84% accuracy and 100% sensitivity in a three-class classification task, demonstrating that carefully optimized CNN architectures can match or exceed more complex models while remaining clinically practical.

### 3.4 Experimental settings

The experiments were performed on Google Colab using Keras deep learning framework. The final dataset was split into a training set and a testing set with a ratio of 0.7:0.3 respectively.

During training, the model is exposed to the data and adjusts its parameters to reduce the difference between the model's predictions and the actual labels in the training data. The model learns to classify the images in the training file by comparing its predictions with the real responses. It then adjusts its parameters to minimize the difference between the model's predictions and the actual labels on the training data, called the loss. After extracting features from the supplied images, the model is then able to predict the categories to which the new images belong, based on the similarities detected with the training examples. The model was trained with a mini-batch size of 4 for 10 epochs. The Stochastic Gradient Descent (SGD) method with a learning rate of 0.001 as optimizer was applied to minimize the loss function and improve the accuracy of the model. The SGD method updates the weights of the neural network during the training.

To avoid over-fitting and measure its ability to classify new images, 15% of the training data was assigned to the validation set. The validation set is used after each epoch to evaluate the model's generalization performance. The error between the predictions and the real labels is calculated using the categorical crossentropy loss function.

Finally, once training is complete, test data are used to evaluate the model's final performance on completely new data, thus guaranteeing its reliability. Model's final classification performance is evaluated through metrics such as accuracy, precision, loss, sensitivity (recall), and specificity. Macro parameters were used to minimize the impact of class imbalance by giving equal weight to the performance of each class.

In a medical context, a high sensitivity is crucial to minimize the false negative and avoid missing a malignant nodule, while a high specificity is important to minimize the false positive and avoid wrong diagnosis.

## 4 Results and discussion

Despite the excellent performance achieved, several limitations must be acknowledged. The private clinical dataset remains relatively small and does not yet represent full-scale prospective validation. However, this deliberate phased approach allowed controlled integration of real-world clinical data while ensuring methodological rigor.

The present work should therefore be considered as an initial translational step toward clinical implementation rather than a definitive deployment. Future studies will focus on expanding the clinical dataset, incorporating multicenter data, and conducting prospective validation studies to further confirm robustness and generalizability. The clinical dataset was used as part of a controlled validation strategy rather than for large-scale training, allowing preliminary assessment under real acquisition conditions.

**Figure 5:**
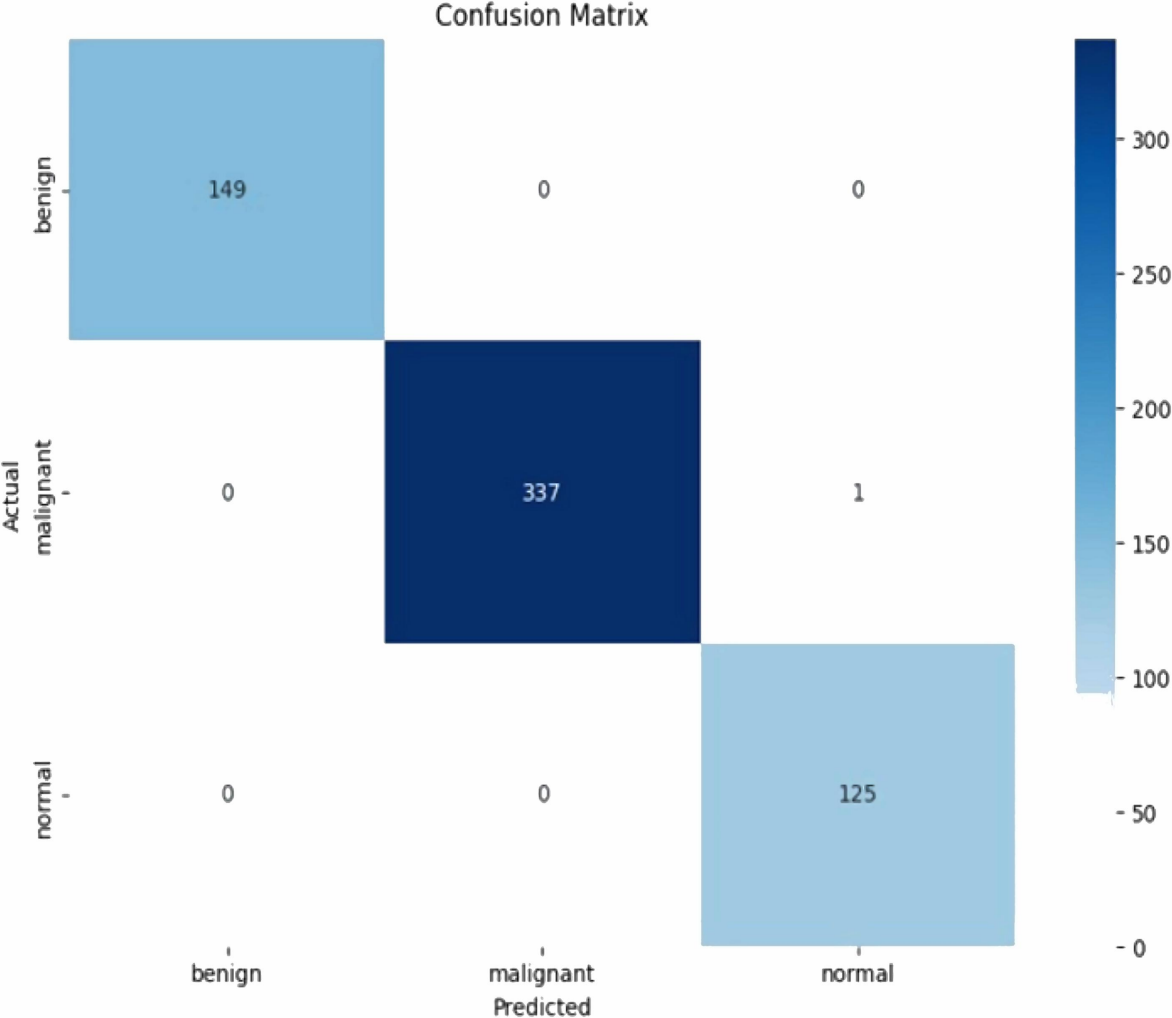
Confusion matrix.

**Figure 6:**
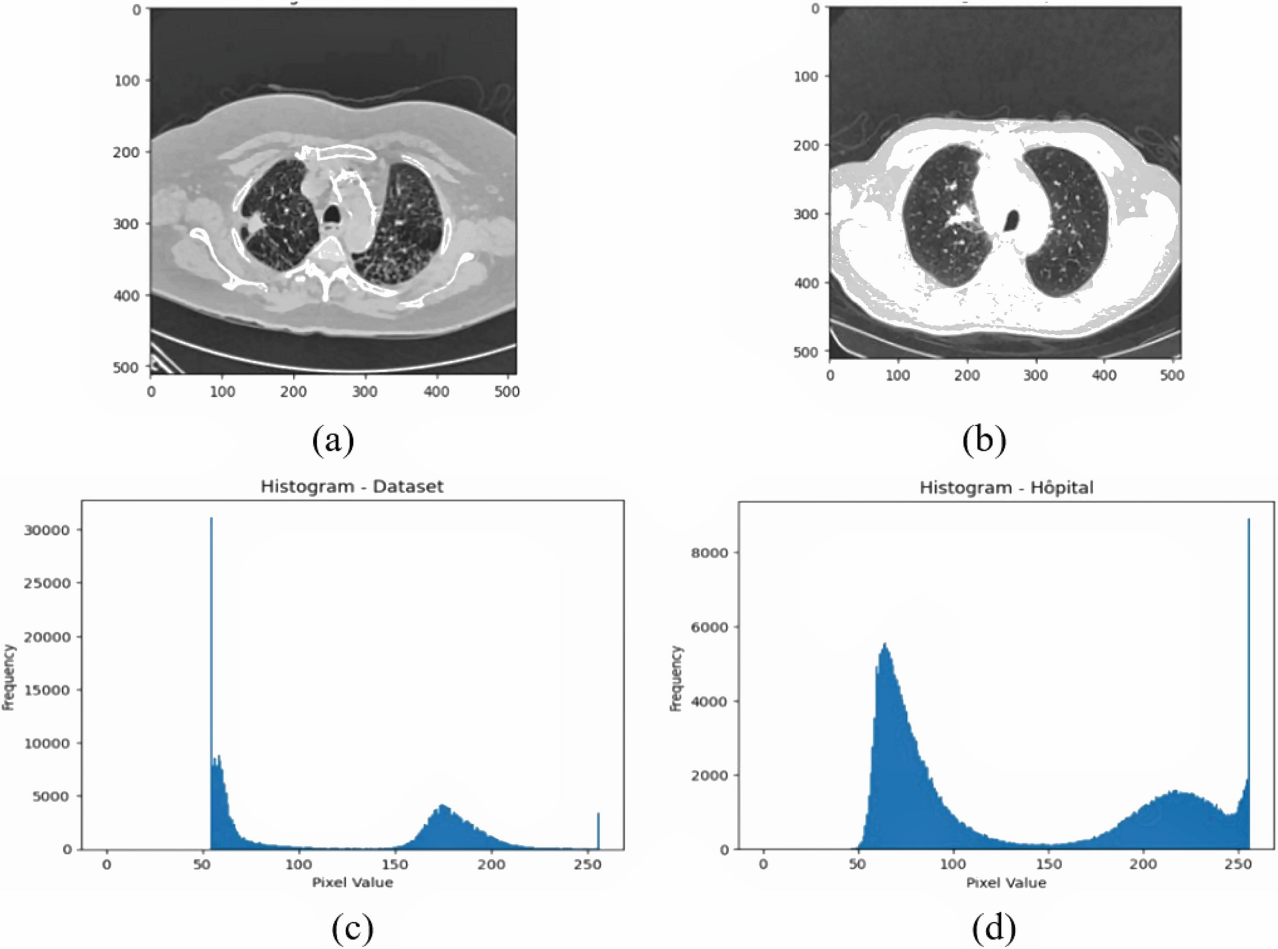
Differences between public dataset images and internal images **(a)** illustrates an image from SPIE-AAPM dataset, and **(c)** corresponds to its histogram. **(b)** illustrates an image from Cheikh Zaid Hospital, and **(d)** corresponds to its histogram. Images are raw, none CLAHE processing is applied.

In the aim of improving the diagnosis of lung nodule, our study turned around a CNN model trained with several database. The results obtained in this study achieved strong classification performance, with an accuracy of 99.84% and a low loss of 0.01. Figure 5 shows the confusion matrix results, and Figure 6 illustrates the differences between public dataset images and internal hospital images while detailed classification results are presented. As depicted in Figure 5, only one image was confused as normal category. Table 3 presents the classification report of our approach based on the confusion matrix.

**Table 3:** The classification report of our proposed approach.

Classes	Precision	Recall	F1-Score
Benign	99.87%	100%	100%
Malignant	99.76%	100%	100%
Normal	99%	100%	100%

Our study demonstrates significant advancements in lung nodule classification through several key achievements. The proposed CNN model achieved exceptional performance metrics of 99.84% accuracy, 99.97% precision, 99.84% sensitivity, and 99.82% specificity, comparing favorably with previous approaches in the literature (Table 4). These results are particularly notable as they were accomplished while addressing the more clinically challenging three-class classification problem (malignant/benign/normal), where most comparable studies focused only on binary classification.

**Table 4:** Comparison of the performance metrics of previous studies and the proposed model.

Approach	Accuracy	Precision	Sensitivity	Specificity
Kareem et al. (10)	89.89%	98.55%	97.14%	97.50%
Al-Yasriy et al. (8)	93.54%	97.1%	95.71%	95.00%
AL-Huseiny et al. (9)	94.38%	–	95.08%	93.7%
Abe et al. (12)	95.43%	–	93.40%	97.09%
Elhassan et al. (13)	96.5%	96.0%	96.2%	–
Elhassan et al. (13)	97.82%	–	–	–
Saechueng et al. (11)	98.32%	98.10%	96.18%	–
Catal Reis et al. (14)	99.55%	98.81%	99.49%	–
Ashames et al. (15)	99.80%	–	–	–
Shatnawi et al. (16)	99%	99.1%	98.4%	–
Shatnawi et al. (16)	100%	99.2%	98.0%	–
Our proposed approach	99.84%	99.97%	99.84%	99.82%

The success of our approach can be attributed to three main methodological choices (Figure 6). First, we developed a comprehensive data integration strategy that combined public datasets (IQ-OTH/NCCD and SPIE-AAPM) with carefully curated clinical images from Cheikh Zaid Hospital. This hybrid approach created a robust training corpus capturing both diverse nodule characteristics and real-world imaging variations, explaining our model's superior generalization capabilities. Second, we established a rigorous clinical validation framework, beginning with a conservative sample size of 51 images from 12 patients to develop and refine our methodology under controlled conditions. Third, our optimized CNN architecture demonstrated that carefully designed networks can outperform more complex hybrid models while maintaining clinical practicality.

### 4.1 Clinical implementation and future work

From a clinical perspective, our model's perfect sensitivity (100%) ensures no malignant cases are missed, while its near-perfect specificity (99.82%) minimizes unnecessary interventions achieving a balance that has eluded previous studies. The current limitations regarding dataset size and nodule diversity represent deliberate, strategic choices in our phased implementation approach rather than shortcomings.

Ongoing work focuses on three key areas: expanding the hospital dataset through our established ethical collection framework, developing nodule-size- invariant classification capabilities, and creating clinician-friendly interpretation interfaces. These efforts build on the strong foundation established by this study's successful integration of research-grade algorithms with real-world clinical data and workflows.

This research provides both a technical benchmark and a validated pathway for transitioning AI diagnostics from research to clinical practice in pulmonary nodule management.

The demonstrated performance, combined with our methodological approach to clinical implementation, addresses critical gaps in the field while opening new opportunities for improving early lung cancer detection and patient outcomes.

## 5 Conclusion

This study presents a high-performance Convolutional Neural Network (CNN) model for pulmonary nodule classification into three clinically relevant categories: malignant, benign, and normal. The model demonstrated outstanding diagnostic performance, achieving an accuracy of 99.84%, sensitivity of 100%, and specificity of 99.9%, establishing a strong benchmark for AI-assisted diagnosis in thoracic imaging. These results stem from an integrative approach combining carefully selected public datasets with real clinical CT images from Cheikh Zaid Hospital, enabling the model to adapt to the variability commonly encountered in routine medical practice.

The clinical implications of this work are significant. The model's perfect sensitivity minimizes the risk of missed malignant nodules, while its high specificity reduces false positives and the likelihood of unnecessary diagnostic procedures. As such, the proposed system shows strong potential as a clinical decision-support tool capable of assisting radiologists, optimizing diagnostic workflows, and improving patient management in lung cancer screening.

Beyond its technical performance, this study represents an important step toward the real-world deployment of AI in radiology. The integration of clinical imaging data demonstrates the feasibility of translating research-level algorithms into practical hospital environments. However, further work is required to confirm clinical impact and generalizability.

Future developments will focus on three main directions: expanding the clinical dataset to improve robustness across diverse populations and imaging conditions, enriching the training data with a wider range of nodule characteristics (size, morphology, and location), and developing user-friendly clinical interfaces accompanied by blinded prospective trials. This work should be interpreted as an early-stage translational study rather than a definitive clinical deployment. Multicenter validation studies will be essential to support large-scale adoption and integration into routine diagnostic workflows. Future work will also include systematic ablation experiments to quantify the contribution of each preprocessing step.

## Data Availability

The datasets presented in this article are not readily available because the clinical dataset from Cheikh Zaid Hospital is subject to restrictions due to patient privacy concerns and ethical regulations. Access to this data is not permitted to protect patient confidentiality, in compliance with HIPAA standards, Moroccan bioethics legislation (Law 28-13), and the ethical oversight of the hospital's Institutional Review Board and the university ethics committee. The public datasets (IQ-OTH/NCCD and SPIE-AAPM) referenced in the study are available from their respective sources, the Kaggle platform and the National Cancer Institute Imaging Data Commons. Requests to access the datasets should be directed to the clinical dataset from Cheikh Zaid Hospital (https://hcz.ma/#contact).
